# Adverse Childhood Experience as a Risk Factor for Developing Type 2 Diabetes among the Jazan Population: A Cross-Sectional Study

**DOI:** 10.3390/children10030499

**Published:** 2023-03-02

**Authors:** Omar Oraibi, Ali T. Ghalibi, Mohammed O. Shami, Meshal J. Khawaji, Khalid A. Madkhali, Abdulrahman M. Yaseen, Sultan M. Hakami, Nirmin H. Alhazmi, Khulud H. Mahla, Marwah A. Qumayri, Khalid A. Majrashi, Abdulrahman Hummadi, Mohammed A. Madkhali, Abdulaziz H. Alhazmi

**Affiliations:** 1 Faculty of Medicine, Jazan University, Jazan 45142, Saudi Arabia; 2 Jazan Endocrinology and Diabetes Center, Ministry of Health, Jazan 82722, Saudi Arabia

**Keywords:** adverse childhood experiences, risk factors, type 2 diabetes mellitus, child abuse, abuse, Saudi Arabia

## Abstract

Background: Adverse Childhood Experiences (ACEs), such as childhood abuse, neglect, and family dysfunction, prevent appropriate emotional, behavioral, and physical development. They are also a major public health issue, and have been debatably linked to chronic diseases, including type 2 diabetes mellitus (T2DM). T2DM is highly prevalent in Saudi Arabia, and various theories have been raised to explain the epidemiology of diabetes. However, few studies have discussed the relationship between ACEs and T2DM. Thus, we aimed to evaluate the association between ACEs and T2DM in Jazan Province, Saudi Arabia. Methods: This observational, cross-sectional study was conducted using a validated questionnaire distributed among patients with T2DM in a diabetes center. The *t*-test and Chi-Square test were used for comparison, and the *p*-value was set at <0.05 for significance. Results: A total of 579 participants were involved in this study, and 303 (52.33%) were female. Among the included participants, 45.25% were diagnosed with T2DM. About 28.71% of participants with diabetes experienced verbal abuse, 16.09% experienced physical abuse, and 30.91% reported that parents beat them. Additionally, 1.58% of participants with diabetes reported living with a family member who abused substances, 8.83% believed that no one would take them to the doctor even if essential, 12.62% of participants with diabetes felt that no one would protect them, and 23.03% reported that they felt no one in their family loved them. All reported ACEs were significantly associated with a high risk of T2DM (*p* < 0.05), and the more frequent the ACEs, the more the risk of T2DM (*p* = 0.0001). Conclusions: This study indicated that ACEs are significantly associated with the development of T2DM, and the risk increases with the frequency of ACEs, which aligns with other studies. Further national studies are required to understand how ACEs could contribute to T2DM, and preventive interventions in childhood must be considered to reduce the burden of T2DM.

## 1. Introduction

Diabetes is a chronic metabolic disease characterized by elevated blood glucose (or blood sugar) levels, leading to serious complications in many organs including the heart, blood vessels, eyes, kidneys, and nerves [1]. Diabetes is classified into many types based on its etiology, and the most common type is type 2 diabetes (T2DM), which is seen in adults, and occurs when the body becomes more resistant to insulin [2]. In the past decades, the prevalence of T2DM has risen dramatically in many countries around the globe and factors contributing to this elevation are obesity, sedentary lifestyles, and unhealthy diets [1,2,3].

Several studies have questioned whether Adverse Childhood Experiences (ACEs), including childhood exposure to physical, sexual, and emotional abuse, neglect, and family dysfunction, could hinder healthy emotional, behavioral, and physical development and could contribute to chronic diseases, including T2DM [3]. ACEs are traumatic events that can be stressful for children and have negative consequences for their neurological, physiological, and social development, especially if repeated. Children cannot access supporting connections or other coping mechanisms [3].

Some studies have found links between early adversity and physical ailments like heart disease, stroke, and chronic respiratory disorders, presumably due to genetic predisposition, induced inflammation, or other processes [4,5,6,7]. It was observed that people with diabetes and ACEs have a greater mortality rate than adults with either diabetes or ACEs alone. In contrast, people with ACEs have greater morbidity rates and are more prone to participate in risky health behaviors, increasing the risk of diabetes [8]. These studies were supported by data from Saudi Arabia, in which Almuneef et al. conducted a national study in 2013 and concluded that ACEs are linked to diabetes mellitus and mental disorders [9].

ACEs bear a significant risk factor for diabetes, and individuals with four or more ACEs have a significantly higher risk (1.6 times) of pre-diabetes and diabetes than those without ACEs [10]. A study conducted in China on middle-aged and old Chinese subjects showed that hunger, unfavored socioeconomic status during childhood, and parental abuse were significantly associated with the development of T2DM and cardiovascular diseases [11]. Another study conducted in Singapore indicated that childhood emotional neglect, parental separation, divorce, the death of a parent, having one or two ACEs, and young age were significantly associated with higher odds of diabetes [12].

The prevalence of diabetes including T2DM in Saudi Arabia is alarming. Factors such as sedentary life, lack of physical activity, high rate of consanguinity, and notional habits have been suggested as important risk factors. However, more studies need to evaluate the influence of ACEs on T2DM in Saudi Arabia. Therefore, this study aimed to evaluate the association between ACEs and T2DM in Jazan Province in southwestern Saudi Arabia. Furthermore, other associated risk factors for T2DM are assessed.

## 2. Materials and Methods

### 2.1. Study Design

This observational, cross-sectional survey was conducted in a diabetes center in Jazan, Saudi Arabia, a region hugely populated with about two million people, and records a high number of patients with diabetes.

### 2.2. Study Tool and Data Collection

In this study, we used a validated questionnaire distributed among patients with T2DM using a link that directed these patients to the digital version of the questionnaire with the help of a continuous data collector. The questionnaire had two parts, and the first included questions on sociodemographic data, such as gender, age, job status, salary, marital status, education levels of parents, having relatives, body mass index (BMI), and residential place. Questions about T2DM diabetes were included with a confirmational diagnostic method as reported by participants. The second part included the Arabic version of ACEs published by the World Health Organization [13]. The questionnaire comes with questions on childhood events related to physical and emotional abuse, a history of violence against household members, or living with someone who a substance abuser is or with mental or psychological problems [13]. 

### 2.3. Sample Size Calculation

This study’s sample size was estimated using the Raosoft sample size calculator (Raosoft Inc., Seattle, WA, USA) (http://www.raosoft.com/samplesize.html, accessed on 15 August 2022). Considering the Jazan region population of about two million, a 95% confidence interval, a 5% margin of error, and a 50% response distribution, the minimum sample size was 385. However, we included 579 participants in our study to increase the significant power of this study. 

### 2.4. Participants’ Inclusion and Exclusion Criteria

We included patients with T2DM and those 18 years or older. Data were collected between September and November 2022, and those diagnosed with diabetes but not T2DM who resided outside of Jazan province, or who refused to participate, were excluded. Patients who visited the diabetes center at the time of data collection and were confirmed not to have T2DM were added for comparison purposes. 

### 2.5. Statistical Analysis

Statistical analysis was run using the Statistical Package for the Social Sciences (SPSS version 23, IB, Chicago, IL, USA). Data were analyzed using descriptive and comparative statistics. Frequencies and percentages were used for the categorical variables, and continuous variables such as age and BMI were analyzed using mean and standard deviation (SD). *T*-tests and Chi-Square tests were used to compare variables. A *p*-value < 0.05 was considered statistically significant.

### 2.6. Ethical Approval

The ethical approval was obtained from the Jazan Health Ethics Committee, Jazan, Saudi Arabia (Permission number 2289, dated 1 September 2022). All participants knew the study’s goals to guarantee complete privacy and confidentiality. They were also permitted to express if they wished to discontinue participation at any moment. Further, data collectors did not collect identifiers or personal information; only the study’s investigators had access to the shared document where the study’s data were kept.

## 3. Results

### 3.1. General Characteristics of the Participants

A total of 579 participants were involved in this study, with a mean age of 40.45 ± 12.93 years. Of all participants, 276 (47.67%) were male, 303 (52.33%) were female, 262 (45.25%) were diagnosed with T2DM, mostly (n = 165, 62.98%) confirmed by HBA1c, and 45.94% had a first-degree relative with T2DM. Most participants (n = 378, 65.28%) were married, employed (n = 314, 54.23%), and with university-level education (n = 422, 72.88%). Regarding the income distribution, 31.61% of participants earned a monthly income of less than 5000 Saudi riyals (SAR), 26.42% of participants earned a monthly income between 5000 and 10,000 SAR, 26.25% of participants earned between 10,000 and 15,000 SAR, and only 15.72% of the participants had a monthly income more than 15,000 SAR. The mean BMI for all participants was 26.97 ± 5.54, 201 (34.72%) were overweight, and 151 (26.08%) were obese. Table 1 shows a detailed description of the general characteristics of the study participants.

### 3.2. Characteristics of the Participants with Type 2 Diabetes Mellitus Compared to Those without Type 2 Diabetes Mellitus

There were 262 participants with T2DM, representing 45.25% of all participants. More than half of them were males (n = 153, 58.40%), married (n = 193, 73.66%), employed (n = 154, 58.78%), educated with university-level degrees (n = 174, 66.41%), overweight BMI (n = 96, 36.64%), and with first-degree relatives with T2DM (n = 121, 46.18%). Of participants without T2DM (n = 317, 54.75%), most were females (n = 194, 61.20%), married (n = 185, 58.36%), employed (n = 160, 50.47%), educated with university-level degrees (n = 248, 78.23%), with normal BMI (n = 107, 33.75%), with monthly income less than 5000 SAR (n = 117, 36.91%), and with first-degree relatives with T2DM (n = 172, 54.26%). Table 2 compares the characteristics of the participants with T2DM and without T2DM. All demographic characteristics significantly (*p* < 0.05) correlated with T2DM, except for having a first-degree relative relationship.

### 3.3. Adverse Childhood Events among Participants with Type 2 Diabetes Mellitus Compared to Those without Type 2 Diabetes Mellitus

Less than a third (28.71%) of participants with T2DM reported experiencing verbal abuse, 16.09% experienced physical abuse, and 30.91% reported being beaten by parents. Additionally, 1.58% of participants with T2DM reported living with a family member who abused substances, 7.89% had a household member with depression or another mental illness, and 2.52% reported having a household member who attempted suicide. Of all participants with T2DM, 8.83% believed that no one would take them to the doctor even if essential, 12.62% felt that no one would protect them, and 23.03% reported that they felt no one in their family loved them. All correlations between ACEs and T2DM were statically significant (*p* = 0.001). Participants reporting ACEs were at a significantly (*p* = 0.001) higher risk of T2DM than those without ACEs. Table 3 shows the answers about ACEs by the participants with T2DM compared to those without T2DM.

### 3.4. Adverse Childhood Events of the Participants with Type 2 Diabetes Mellitus Compared to Those without Type 2 Diabetes Mellitus

**Figure 1:**
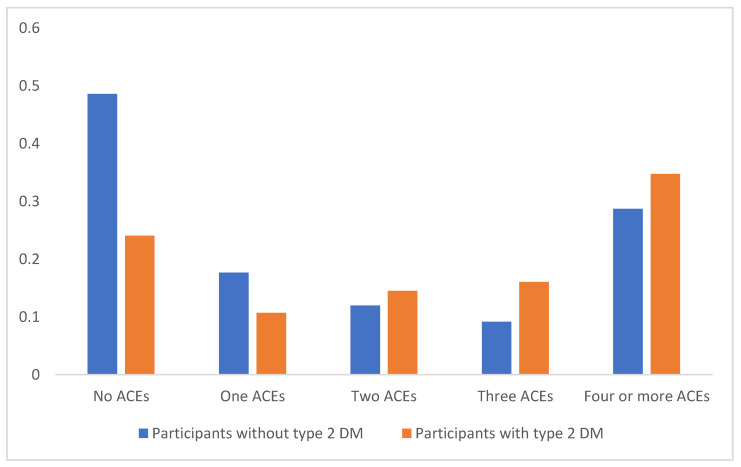
Association between ACEs and T2DM.

Figure 1 shows the association between ACEs and T2DM; the more ACEs, the greater the odds, and experiencing four or more ACEs was significantly associated with higher odds of diabetes (*p* = 0.0001). Participants without ACEs had significantly lower odds of T2DM (*p* = 0.0001).

## 4. Discussion

This study evaluated the association between ACEs and T2DM, a relationship that has been debated. Very few studies have discussed this relationship in Saudi Arabia, and therefore we aimed to assess this relationship among adults with T2DM in the Jazan region in Saudi Arabia. This study aligns with another study exploring ACEs, mental health, and risky behaviors among Saudis [14]. We found a significant association between ACEs and T2DM, and the risk of T2DM increased with the frequency of ACEs, which agrees with another study that showed that one or more ACEs were associated with a higher risk of T2DM among individuals living in the central region of Saudi Arabia [8]. Similarly, a systematic literature review and meta-analysis showed that ACEs, particularly childhood neglect, family dysfunction, and two or more ACEs, are associated with a higher risk of T2DM [15]. It was found that childhood economic adversity and physical, sexual, and verbal abuse were associated with T2DM [16,17]. On the other hand, a systematic literature review by Zhu et al. [16] reported that emotional abuse, domestic violence, parental divorce, parental death, neglect, and living with a family member with substance abuse were not significantly associated with diabetes. It is noteworthy that the sample size in these studies is different from our study sample, and the explanation for these variations might be related to the heterogeneity of the population and the high risk of recall bias in the previous studies. 

It was proposed by a 30-year follow-up study using court-recorded data conducted by Widom et al. that childhood physical abuse and neglect are associated with T2DM later in middle adulthood [18]. Physical and sexual abuse in childhood and adolescence were also linked to increased T2DM risk in adult women, supporting Monnat and Chandler’s data [18,19]. Sex has been suggested as another risk factor, and our study found that women with ACEs were at higher risk of T2DM than their male counterparts, which might be due to the higher incidence of ACEs in women or other factors that should be sought. As reported in the current study, studies on sex differences in the profiles of ACEs found that compared to men, women reported a more complex history of ACEs [20,21]. Further, women’s mental health, social functioning, and emotional stability are more negatively impacted by exposure to ACEs compared to men, with family dysfunction as a major poor social outcome [21]. Another study evaluating the relationship between ACEs and T2DM among women showed a strong relationship between excess weight, obesity, and diabetes [22]. The link between ACEs and the development of T2DM and other chronic physical problems has been explained by many theories. ACEs disrupt neurodevelopment, particularly the hypothalamic-pituitary-adrenal axis (HPA) [23]. Dysregulation of HPA has been suggested to be a pre-diabetes condition for people with a high risk of T2DM [23,24]. Moreover, it raises blood triglycerides, free fatty acids, and inflammatory markers, which exposes them to depression and other chronic disease risks, and upregulates gluconeogenesis, causing hyperglycemia [11,23]. Previous studies have suggested that stress and inflammatory markers may explain the link between diabetes and depression [25], and other studies have even suggested that ACEs could be associated with biological risk already present at an early age, which appeared to cause physiological changes that might be associated with later development of diseases [26]. Therefore, ACEs are indirectly linked to a higher risk of T2DM through their effects on mental health, especially their link to depression and low quality of life, which were identified as risk factors of T2DM [11,25,27]. A cohort study showed that ACEs were associated with a high risk of T2DM, with the odds of diabetes increasing by almost 11% for each added ACE [26]. ACEs were also found to be indirectly related to T2DM through cardiometabolic dysregulation in addition to depression [27].

In a pilot study conducted in Riyadh, Saudi Arabia, the authors found that most participants reported experiencing one or more ACEs, and almost a third of the participants had experienced four or more ACEs [28]. Another study conducted to evaluate the prevalence and connection of ACEs with both physical and mental diseases in eastern Saudi Arabia found that the majority of study participants (81.8%) had experienced four or more ACEs, with emotional neglect being the most prevalent ACE type (82.2%) [29]. Additionally, women with four or more ACEs were at higher risk of insomnia, stress, and depression, which aligns with other previous studies [20,21]. Compared to those with only one ACE, those with four or more ACEs are more likely to experience physical diseases [29]. Studies conducted in Saudi Arabia comparing people with ACEs to those without ACEs revealed a two-fold greater risk of physical health problems such as hypertension, T2DM, coronary heart disease, and obesity among people with ACEs [13,30,31], agreeing with another study conducted in Saudi Arabia [19]. This high prevalence of ACEs in Saudi Arabia might contribute to high rates of T2DM in Saudi Arabia, as supported by the WHO ranking, which puts Saudi Arabia in second place among Middle Eastern countries with a high rate of diabetes and seventh highest worldwide [32,33]. Further, a recent study evaluating the prevalence of T2DM in Saudi Arabia from 1999 to 2022 estimated it to be 29.2% in 2011 and 44.1% in 2022 [34]. These reports indicate that diabetes is a huge health burden in Saudi Arabia, and future strategies, programs, and research should consider ACEs as a factor in reducing the burden.

It is noteworthy that this study was conducted just a few months after the alleviation of the COVID-19 pandemic restrictions in Saudi Arabia, a period that had social and psychological consequences on most countries around the globe, including Saudi Arabia and the Jazan region [35]. Thus, the results of the current studies should be interpreted carefully as COVID-19 consequences may have resulted in biases related to the reported information. However, the association between ACEs and the development of T2DM in this study cannot be ignored, and this should encourage local health officials to conduct a larger study that may include other chronic diseases.

When interpreting and generalizing the results of the current study, some limitations should be considered. The study’s cross-sectional design could not explain the temporal relationship between the study variables. Unlike some previous studies [36], different types of ACEs, such as the correlation of sexual abuse between ACEs, physical health, and diet, were not investigated in our study. However, we studied alcohol and substance abuse, which was not previously studied in Saudi Arabia [13], and our sample was homogeneous, increasing the accuracy of our results. We recommend further longitudinal, prospective clinical studies with an extensive and representative sample to understand the association between ACEs and T2DM [37,38].

## 5. Conclusions

This study confirmed that T2DM is associated with increasing ACEs in Jazan, Saudi Arabia, and more ACEs are significantly associated with a higher risk of T2DM development, highlighting the need to reduce the risk variables linked to ACEs. Since parents and other family members are primarily in charge of raising children, establishing education programs aimed at reducing child abuse and improving child well-being by targeting them might be an effective intervention. Therefore, the findings of this study can be utilized to fine-tune public awareness campaigns that target all families in Jazan and encourage healthy behaviors among youngsters. Further, larger studies based on medical record data and including other chronic diseases could be conducted for a better understanding of the consequences of ACEs at an individual and public level, and how preventive interventions in childhood could be tailored to limit the burden of T2DM.

## Figures and Tables

**Table 1:** General characteristics of the participants (n = 579).

Age in Years	Mean|SD	40.45	12.93
Sex	Female	303	52.33%
	Male	276	47.67%
Marital status	Single	148	25.56%
	Married	378	65.28%
	Divorced	37	6.39%
	Widow	16	2.76%
Job	Student	95	16.41%
	Employed	314	54.23%
	Looking for job	80	13.82%
	Retired	90	15.54%
Monthly income in SAR	Less than 5 k	183	31.61%
	from 5 k to 10 k	153	26.42%
	From 10 k to 15 k	152	26.25%
	More than 15 k	91	15.72%
Residence	City	180	31.09%
	Governate	188	32.47%
	Village	192	33.16%
	Mountain	19	3.28%
Educational level	Informal education	10	1.73%
	General education	130	22.45%
	University level	422	72.88%
	Postgraduate	17	2.94%
Father	Informal education	138	23.83%
	General education	237	40.93%
	University level	189	32.64%
	Postgraduate	15	2.59%
Mother	Informal education	261	45.08%
	General education	177	30.57%
	University level	138	23.83%
	Postgraduate	4	0.69%
BMI classification	Underweight	32	5.53%
	Normal	195	33.68%
	Overweight	201	34.72%
	Obese	151	26.08%
First degree relative	No	313	54.06%
	Yes	266	45.94%
Have you been diagnosed with type 2 DM	No	317	54.75%
	Yes	262	45.25%
BMI	Mean | SD	26.97	5.54
How was your diagnosis first confirmed	FBG	63	24.05%
	RBG	30	11.45%
	HBa1c	165	62.98%
	OGT	4	1.53%

SD: Standard deviation. BMI: Body mass index. SAR: Saudi Riyals. FBG: Fasting blood glucose. RBG: Random blood glucose. HBA1c: Hemoglobin A1c. DM: Diabetes mellitus.

**Table 2:** Characteristics of the participants with type 2 diabetes mellitus compared to those without type 2 diabetes mellitus.

Variables		Participants without T2DM (n = 317, 54.75%)		Participants with T2DM (n = 262, 45.25%)		*p*-Value
Age in years (mean|SD)		35.95	11.57	44.34	12.80	0.0001 *
Sex	Female	194	61.20%	109	41.60%	0.0001 *
	Male	123	38.80%	153	58.40%	
Marital status	Single	117	36.91%	31	11.83%	0.0001 *
	Married	185	58.36%	193	73.66%	
	Divorced	11	3.47%	26	9.92%	
	Widow	4	1.26%	12	4.58%	
Job	Student	79	24.92%	16	6.11%	0.0001 *
	Employed	160	50.47%	154	58.78%	
	Looking for job	57	17.98%	23	8.78%	
	Retired	21	6.62%	69	26.34%	
Monthly income in SAR	Less than 5 k	117	36.91%	66	25.19%	0.007 *
	from 5 k to 10 k	69	21.77%	84	32.06%	
	From 10 k to 15 k	82	25.87%	70	26.72%	
	More than 15 k	49	15.46%	42	16.03%	
Residence	City	101	31.86%	79	30.15%	0.703
	Governate	104	32.81%	84	32.06%	
	Village	104	32.81%	88	33.59%	
	Mountain	8	2.52%	11	4.20%	
Educational level	Informal education	2	0.63%	8	3.05%	0.0001 *
	General education	51	16.09%	79	30.15%	
	University level	248	78.23%	174	66.41%	
	Postgraduate	16	5.05%	1	0.38%	
Fathers’ educational level	Informal education	54	17.03%	84	32.06%	0.0001 *
	General education	133	41.96%	104	39.69%	
	University level	116	36.59%	73	27.86%	
	Postgraduate	14	4.42%	1	0.38%	
Mother’s educational level	Informal education	110	34.70%	151	57.63%	0.0001 *
	General education	107	33.75%	70	26.72%	
	University level	98	30.91%	40	15.27%	
	Postgraduate	2	0.63%	1	0.38%	
BMI classification	Underweight	28	8.83%	4	1.53%	0.002 *
	Normal	107	33.75%	88	33.59%	
	Overweight	105	33.12%	96	36.64%	
	Obese	77	24.29%	74	28.24%	
First-degree relative relationship	No	172	54.26%	141	53.82%	0.933
	Yes	145	45.74%	121	46.18%	

DM: Diabetes mellitus. SD: Standard deviation. BMI: Body mass index. SAR: Saudi Riyals. * Significant when the alpha criterion for *p*-value was set to 0.05.

**Table 3:** Answers of ACE by the participants with type 2 diabetes mellitus compared to those without type 2 diabetes mellitus.

Variables		Participants without Type 2 DM (n = 317, 54.75%)		Participants with Type 2 DM (n = 262, 45.25%)		*p*-Value
Did a parent or other adult in the household often swear at you, insult you, put you down, or humiliate you?	No	226	71.29%	124	47.33%	0.0001 *
	Yes	91	28.71%	138	52.67%	
Did a parent or other person in the home often threaten you physically?	No	266	83.91%	183	69.85%	0.0001 *
	Yes	51	16.09%	79	30.15%	
Have you often felt that no one in your family loves you?	No	244	76.97%	166	63.36%	0.0001 *
	Yes	73	23.03%	96	36.64%	
Have you often felt that you have no one to protect you?	No	277	87.38%	198	75.57%	0.0001 *
	Yes	40	12.62%	64	24.43%	
Have you often felt that you have no one to take you to the doctor even though you need to?	No	289	91.17%	186	70.99%	0.0001 *
	Yes	28	8.83%	76	29.01%	
Has a parent or someone ever hit you often?	No	219	69.09%	123	46.95%	0.0001 *
	Yes	98	30.91%	139	53.05%	
Have you lived with someone in your family who suffers from drinking or alcohol addiction?	No	312	98.42%	235	89.69%	0.0001 *
	Yes	5	1.58%	27	10.31%	
Have you had a friend who suffers from drinking or alcohol addiction?	No	310	97.79%	233	88.93%	0.0001 *
	Yes	7	2.21%	29	11.07%	
Was a household member depressed or mentally ill	No	292	92.11%	200	76.34%	0.0001 *
	Yes	25	7.89%	62	23.66%	
Did a household member attempt suicide?	No	309	97.48%	230	87.79%	0.0001 *
	Yes	8	2.52%	32	12.21%	
Did you live with your parents	No	33	10.41%	52	19.85%	0.002 *
	Yes	284	89.59%	210	80.15%	
Did your family take care of each other?	No	23	7.26%	54	20.61%	0.0001 *
	Yes	294	92.74%	208	79.39%	

ACE: Adverse childhood experience. DM: Diabetes mellitus. SD: Standard deviation. * Significant when the alpha criterion for *p*-value was set to 0.05.

## Data Availability

Data are available upon a reasonable request from the corresponding author.

## References

[1] Ahmed A.S., Alotaibi W.S., Aldubayan M.A., Alhowail A.H., Al-Najjar A.H., Chigurupati S., Elgharabawy R.M. (2021). Factors Affecting the Incidence, Progression, and Severity of COVID-19 in Type 1 Diabetes Mellitus. BioMed Res. Int..

[2] Smushkin G., Vella A. (2010). What is type 2 diabetes?. Medicine.

[3] Hughes K., Ford K., Bellis M. (2020). Adverse Childhood Experiences (ACEs) and Diabetes|A Brief Review. Public Health Wales. https://research.bangor.ac.uk/portal/files/37959822/PHWBangor_ACEs_Diabetes_Factsheet_5_.pdf.

[4] Ayoub C.C., O’Connor E., Rappolt-Schlichtmann G., Fischer K.W., Rogosch F.A., Toth S.L., Cicchetti D. (2006). Cognitive and emotional differences in young maltreated children: A translational application of dynamic skill theory. Dev. Psychopathol..

[5] Kim-Spoon J., Cicchetti D., Rogosch F.A. (2013). A Longitudinal Study of Emotion Regulation, Emotion Lability-Negativity, and Internalizing Symptomatology in Maltreated and Nonmaltreated Children. Child Dev..

[6] Felitti V.J., Anda R.F., Nordenberg D., Williamson D.F., Spitz A.M., Edwards V., Marks J.S. (1998). Relationship of Childhood Abuse and Household Dysfunction to Many of the Leading Causes of Death in Adults. Am. J. Prev. Med..

[7] Huang H., Yan P., Shan Z., Chen S., Li M., Luo C., Gao H., Hao L., Liu L. (2015). Adverse childhood experiences and risk of type 2 diabetes: A systematic review and meta-analysis. Metabolism.

[8] Almuneef M., Hollinshead D., Saleheen H., AlMadani S., Derkash B., AlBuhairan F., Al-Eissa M., Fluke J. (2016). Adverse childhood experiences and association with health, mental health, and risky behavior in the kingdom of Saudi Arabia. Child Abuse Negl..

[9] Campbell J.A., Mosley-Johnson E., Garacci E., Walker R.J., Egede L.E. (2019). The co-occurrence of diabetes and adverse childhood experiences and its impact on mortality in US adults. J. Affect. Disord..

[10] Lynch L., Waite R., Davey M.P. (2013). Adverse Childhood Experiences and Diabetes in Adulthood: Support for a Collaborative Approach to Primary Care. Contemp. Fam. Ther..

[11] Zhang K., Wu B., Zhang W. (2022). Adverse childhood experiences in relation to comorbid cardiovascular diseases and diabetes among middle-aged and old adults in China. Geriatr. Gerontol. Int..

[12] Subramaniam M., Abdin E., Vaingankar J.A., Chang S., Sambasivam R., Jeyagurunathan A., Seow L.S., Van Dam R., Chow W.L., Chong S.A. (2021). Association of adverse childhood experiences with diabetes in adulthood: Results of a cross-sectional epidemiological survey in Singapore. BMJ Open.

[13] Adverse Childhood Experiences International Questionnaire (ACE-IQ). https://cdn.who.int/media/docs/default-source/documents/child-maltreatment/ace-iq-guidance-for-analysing.pdf?sfvrsn=adfe12bb_2.

[14] Alhowaymel F.M., Alenezi A. (2022). Adverse Childhood Experiences and Health in Rural Areas of Riyadh Province in Saudi Arabia: A Cross-Sectional Study. Healthcare.

[15] Zhang Y., Yin Y., Zhang X., Ye J., Zhang J. (2022). Association of adverse childhood experiences with diabetes: A systematic review and meta-analysis. J. Diabetes Its Complicat..

[16] Zhu S., Shan S., Liu W., Li S., Hou L., Huang X., Liu Y., Yi Q., Sun W., Tang K. (2022). Adverse childhood experiences and risk of diabetes: A systematic review and meta-analysis. J. Glob. Health.

[17] Amemiya A., Fujiwara T., Shirai K., Kondo K., Oksanen T., Pentti J., Vahtera J. (2019). Association between adverse childhood experiences and adult diseases in older adults: A comparative cross-sectional study in Japan and Finland. BMJ Open.

[18] Widom C.S., Czaja S.J., Bentley T., Johnson M.S. (2012). A prospective investigation of physical health outcomes in abused and neglected children: New findings from a 30-year follow-up. Am. J. Public Health.

[19] Monnat S.M., Chandler R.F. (2015). Long Term Physical Health Consequences of Adverse Childhood Experiences. Sociol. Q..

[20] Cavanaugh C.E., Petras H., Martins S.S. (2015). Gender-specific profiles of adverse childhood experiences, past year mental and substance use disorders, and their associations among a national sample of adults in the United States. Soc. Psychiatry Psychiatr. Epidemiol..

[21] Haahr-Pedersen I., Perera C., Hyland P., Vallières F., Murphy D., Hansen M., Spitz P., Hansen P., Cloitre M. (2020). Females have more complex patterns of childhood adversity: Implications for mental, social, and emotional outcomes in adulthood. Eur. J. Psychotraumatol..

[22] Lown E.A., Lui C.K., Karriker-Jaffe K., Mulia N., Williams E., Ye Y., Li L., Greenfield T.K., Kerr W.C. (2019). Adverse childhood events and risk of diabetes onset in the 1979 National longitudinal survey of youth cohort. BMC Public Health.

[23] Kalmakis K.A., Meyer J.S., Chiodo L., Leung K. (2015). Adverse childhood experiences and chronic hypothalamic–pituitary–adrenal activity. Stress.

[24] Mosili P., Mkhize B.C., Ngubane P., Sibiya N., Khathi A. (2020). The dysregulation of the hypothalamic-pituitary-adrenal axis in diet-induced prediabetic male Sprague Dawley rats. Nutr. Metab. (Lond.).

[25] Bădescu S.V., Tătaru C., Kobylinska L., Georgescu E.L., Zahiu D.M., Zăgrean A.M., Zăgrean L. (2016). The association between Diabetes mellitus and depression. J. Med. Life.

[26] Soares S., Rocha V., Kelly-Irving M., Stringhini S., Fraga S. (2021). Adverse Childhood Events and Health Biomarkers: A Systematic Review. Front. Public Health.

[27] Deschênes S.S., Graham E., Kivimäki M., Schmitz N. (2018). Adverse Childhood Experiences and the Risk of Diabetes: Examining the Roles of Depressive Symptoms and Cardiometabolic Dysregulations in the Whitehall II Cohort Study. Diabetes Care.

[28] Al-Eissa M.A., AlBuhairan F.S., Qayad M., Saleheen H., Runyan D., Almuneef M. (2015). Determining child maltreatment incidence in Saudi Arabia using the ICAST-CH: A pilot study. Child Abuse Neglect.

[29] AlHemyari A.H., Al-Zamil N.A., Shaikh A.Y., Al-Eidi D.A., Al-Dahlan H.W., Al-Shamekh S.S. (2022). Prevalence of adverse childhood experiences and their relationship to mental and physical illnesses in the Eastern Region of Saudi Arabia. Brain Behav..

[30] Alhowaymel F. (2020). Geographical Disparity of Adverse Childhood Experiences and Chronic Diseases in Saudi Arabia. Ph.D. Thesis.

[31] Herzog J.I., Schmahl C. (2018). Adverse Childhood Experiences and the Consequences on Neurobiological, Psychosocial, and Somatic Conditions Across the Lifespan. Front. Psychiatry.

[32] Robert A., Al Dawish M., Braham R., Musallam M., Al Hayek A., Al Kahtany N. (2016). Type 2 Diabetes Mellitus in Saudi Arabia: Major Challenges and Possible Solutions. CDR.

[33] Itumalla R., Kumar R., Tharwat Elabbasy M., Perera B., Torabi M.R. (2021). Structural Factors and Quality of Diabetes Health Services in Hail, Saudi Arabia: A Cross-Sectional Study. Healthcare.

[34] Al-Quwaidhi A.J., Pearce M.S., Sobngwi E., Critchley J.A., O’Flaherty M. (2014). Comparison of type 2 diabetes prevalence estimates in Saudi Arabia from a validated Markov model against the International Diabetes Federation and other modelling studies. Diabetes Res. Clin. Pract..

[35] Albeladi F.I., Alluli M.M., Daghriri K.A., Almalki Y.H., Wafi M.Y., Otaif F.A., Sulays Z.Y., Hakami A.A., Alharbi A.A., Alhazmi A.H. (2021). Level of Adherence to COVID-19 Preventive Measures Among HealthCare Workers in Saudi Arabia. Cureus.

[36] Aquilina S.R., Shrubsole M.J., Butt J., Sanderson M., Schlundt D.G., Cook M.C., Epplein M. (2021). Adverse childhood experiences and adultdiet quality. J. Nutr. Sci..

[37] Alhazmi A., Sane F., Lazrek M., Nekoua M.P., Badia-Boungou F., Engelmann I., Alidjinou E.K., Hober D. (2020). Enteroviruses and Type 1 Diabetes Mellitus: An Overlooked Relationship in Some Regions. Microorganisms.

[38] Hurissi E., Alameer M., Ageeli F., Allami M., Alharbi M., Suhail H., Albeishy H., Oraibi O., Somaili M., Hummadi A. (2022). The Association between SARS-CoV-2 Infection and Diabetic Ketoacidosis in Patients with New-Onset Diabetes: A Retrospective Study from a Diabetic Center in Saudi Arabia. Pediatr. Rep..

